# Progress in Fevers

**Published:** 1903-07-11

**Authors:** 


### PROGRESS IN FEVERS.

(*Continued from page* 246.)

**Paratyphoid Fever.**—N. E. Brill[6] gives a summary of 62 cases of this affection published by various observers, including 17 probable cases of his own published in 1897 in which bacteriological examination was imperfect although the absence of bacillus typhosus was demonstrated in fæces, urine, and aspirated splenic blood. The first cases of paracolon or paratyphoid infection in man were reported in France in 1896 by Achard and Bensaude. Since then cases have been reported by Gwyn, Cushing, Schottmüller, and many others, and Feyfer and Kayser have recently reported an outbreak of 14 cases at Eibergen, in Holland. Brill divides the cases into two groups. One, the commoner type, shows the usual features of typhoid fever infection, the other begins more abruptly with more marked gastro-intestinal symptoms and less typical fever. Special points of distinction from typhoid which he emphasises are:—the shorter duration of the disease—an average of a little over two weeks, although the cases may be much more prolonged; a tendency for the fever to terminate by crisis or by rapid lysis; the marked early prostration; and slight tendency to dicrotism of the pulse. Cases can, however, be only absolutely differentiated by agglutination and cultural tests. Coleman[7] says one can only rely upon the "absence of the Widal reaction in proper dilution (not less than 1 in 40) with a positive reaction against a known paratyphoid bacillus, or the recovery of a paratyphoid bacillus from the blood, urine, stools, or complicating inflammatory process." Kayser and Schottmüller[8] divide the organisms found in these cases into two groups, type A and type B, which appear to correspond to the "paratyphoid" and "paracolon" groups respectively. Both groups produce gas in glucose media, but do not ferment lactose, coagulate milk, nor form indol, and only agglutinate typhoid sera imperfectly in low dilutions. The paratyphoid group "interact to agglutination tests without exception," but the paracolon group do not show mutual reactions. Both groups produce an initial short acidity in milk and whey, but in the paracolon group this is soon replaced by alkalinity. The "paracolons" also produce gas more freely in glucose media and retain the yellow colour produced by growth on neutral red agar. The "paratyphoids" ferment glucose less freely and the yellow colour produced by their growth on agar changes again to red. H. E. Allen[9] reports three new cases of this infection. In one, Widal's reaction, and the serum test with a paracolon bacillus were both negative, but the pus from a suppurative cholecystitis, which occurred during convalescence, gave a pure culture of a paracolon bacillus. In the second, complicated by thrombosis of the femoral vein and by cystitis, an actively motile paracolon bacillus was isolated from the blood and the urine. In the third case, the serum reacted with cultures of the bacilli found in cases one and two at dilutions of 1 in 50. The Widal reaction was sometimes obtained in dilution of 1 in 10. Allen has collected three fatal cases with autopsies, those of Berg and Libman, of Strong, and of Longcope. The first case was probably a mixed typhoid and paracolon infection. Strong's case was incomplete and complicated by malaria, Longcope's case showed only the lesions of an acute toxæmia, viz., enlarged spleen, cloudy swelling of liver and kidneys, and focal necroses in the liver. Both intestines were practically normal, and the mesenteric lymph glands were not swollen. But although absence of intestinal ulceration or affection of the intestinal lymph nodes has been reported altogether in three cases, the occurrence in several reported cases of intestinal hæmorrhage suggests that intestinal lesions do occur. Some reported cases of typhoid fever without intestinal lesion may be examples of para-typhoid fever. The complications of this disease are very numerous and frequent. They include bronchitis, pneumonia, pleurisy, endocarditis, thrombosis, cerebral embolism, phlebitis, meningitis, peritonitis, cholecystitis, nephritis, cystitis, orchitis, furuncles, osteomyelitis, myositis, and suppurative arthritis.

**Typhoid Fever.**—R. M. Le H. Cooper[10] enters at considerable length into the sanitary and hygienic measures adopted by himself during the South African war at Harrismith for the prevention of enteric fever and dysentery. Tables are given showing a very decided decrease in the incidence of locally contracted cases and in mortality in 1902 as compared with 1901. The water supply here appears to have been good and above suspicion. Infection was thought to be carried by flies through the medium of infected excreta. The preventive measures employed in 1902, and carried out in the face of considerable difficulties, were based on this assumption, and were directed to the destruction of flies, the protection of food from flies, and the disinfection and safe disposal of urinary and fæcal excreta. Professor Koch,[11] in a lecture delivered at Berlin on the suppression of typhoid fever, points to the heavy death-roll still caused by this disease in peace and war. Acknowledging the excellence of the doctrines of cleanliness, pure water, and good sanitation, he points out the difficulty of ensuring these, in country districts at any rate, and urges that typhoid fever should be treated on the analogy of cholera. When a case of cholera is suspected we detect the germ, isolate the patient, and disinfect the excreta. Two things are needful, the easy and certain discovery of the infective material and its destruction. Contrary to his former belief, Professor Koch now considers that the vitality of the typhoid bacillus in water and in soil, unless the circumstances are exceptionally favourable, is short-lived, and that instances of prolonged infection from one source are due to fresh infections of that source. The problem of early diagnosis is the first to be solved. For this purpose Widal's reaction sometimes fails, and always appears inconveniently late. The method of bacteriological culture recently elaborated by Drigalski and Conradi is considered the best by Koch. For this method a culture medium has been devised which has a restraining effect on the growth of intestinal bacteria other than those of the coli group. If growths of cocci and other organisms are formed they can be distinguished by the appearance of their colonies. To distinguish between the colonies of typhoid and coli organisms the nutrient medium is tinged with litmus solution. Colon bacilli make strong acid-forming colonies; those of bacillus typhosus show an alkaline reaction in this culture medium. The serum-agglutination test is used to confirm the presence of typhoid colonies. In this way Koch finds he can give an absolutely reliable diagnosis in from twenty to twenty-four hours, and he has recognised the presence of the bacillus in the stools of patients as early as the second day. The test is also useful for discovering bacilli in the evacuations of "contacts" and "suspects" in whom symptoms are trivial or absent. Koch used this method successfully in stamping out an epidemic in the country district of Trier, where it revealed many unsuspected cases, and where "all the cases were traceable to direct infection from person to person." Isolation and disinfection can in this way be applied very early in any case. The outbreak of enteric fever in Winchester, Southampton, and Portsmouth,[12] which led to the death of the Dean of Winchester and other prominent men, was attributed to sewage-contaminated oysters. Since Sir Charles Cameron pointed out this possible source of typhoid fever in 1880 various reports on the subject have been presented, but no satisfactory attempts at legislation have been carried through. Mussels and cockles are also implicated. Following the Winchester outbreak the Fishmongers' Company[13] took prompt measures to check the sale in London of oysters from contaminated beds.

[6] Med. Rec., Nov. 29. [7] Med. Chron., Dec. [8] Lancet, Feb. 28' p. 602. [9] Amer. Jour. Med. Sci., Jan. [10] Lancet, March 7. [11] Brit. Med. Jour., Feb. 28, p. 503. [12] Ibid., Jan. 3, p. 33. [13] Ibid., Feb. 7, p. 325.

*(To be continued.)*

